# Egyptian Association of Vascular Biology and Atherosclerosis (EAVA) consensus on the usage of proprotein convertase subtilisin/kexin type 9 (PCSK9) inhibitors

**DOI:** 10.1186/s43044-020-00058-0

**Published:** 2020-05-18

**Authors:** Ashraf Reda, Ahmed Shawky Elserafy, Elsayed Farag, Tamer Mostafa, Nabil Farag, Atef Elbahry, Osama Sanad, Ahmed Bendary, Ahmed Elkersh, Mohammed Selim, Morad Beshay, Hazem Khamis

**Affiliations:** 1 Cardiology Department, Faculty of Medicine, Menofia University, Menofia, Egypt; 2 Cardiology Department, Faculty of Medicine, Ain Shams University, Cairo, Egypt; 3 Cardiology Department, Faculty of Medicine, Zagazig University, Zagazig, Egypt; 4 Cardiology Unit, Port Fouad Centre, Port Fouad, Egypt; 5 Cardiology Department, Faculty of Medicine, Benha University, Benha, Egypt; 6 National Heart Institute, Giza, Egypt; 7 Cardiology Department, Faculty of Medicine, 6th of October University, Cairo, Egypt

**Keywords:** Egypt, Dyslipidemia, PCSK-9

## Abstract

**Background:**

The current expert view of the PCSK9 inhibitors’ use in Egypt is still ambiguous.

**Main body:**

Hyperlipidemia is an important, if not the most important, risk factor for the occurrence of atherosclerosis worldwide. Egypt is the most populous country in the Middle East and North Africa and has > 15% of the cardiovascular deaths in the region. The burden of dyslipidemia as seen in the recently published CardioRisk project conducted throughout Egypt shows a high prevalence of dyslipidemia as a risk factor that is still reaching up to 71% in female participants. Reaching the targets for LDL lowering, and thus control of hyperlipidemia, is quite often very difficult especially with the update of the last ESC guidelines. With the advent of PCSK9 inhibitors, the control rate of patients, reduction of cardiac major adverse events, and mortality have been improved. However, Egypt is not considered a rich country on the grounds of annual income, and this raises a concern on which patients would benefit from these expensive medications. Revising the randomized control trials, we analyzed the data that would enable us to control LDL in those patients, at risk, to obtain simple clear indications for the use of these rather expensive medications.

**Conclusion:**

We recommend the use of PCSK9 inhibitors in addition to statins ± ezetimibe in patients with ASCVD, by definition at very high risk; patients with ASCVD at very high risk who do not tolerate appropriate doses of at least three statins; and familial hypercholesterolaemia patients with clinically diagnosed ASCVD, at very high cardiovascular risk.

## Background

Egypt is the most populous country in the Middle East and North Africa and has > 15% of the cardiovascular deaths in the region. The burden of dyslipidemia as seen in the recently published CardioRisk project conducted throughout Egypt shows a high prevalence of dyslipidemia as a risk factor that is still reaching up to 71% in female participants. Reaching the targets for LDL lowering, and thus control of hyperlipidemia, is quite often very difficult especially with the update of the last ESC guidelines. With the advent of PCSK9 inhibitors, the control rate of patients, reduction of cardiac major adverse events, and mortality have been improved. However, Egypt is not considered a rich country on the grounds of annual income, and this raises a concern on which patients would benefit from these expensive medications. Accordingly, the Egyptian Association of Vascular Biology and Atherosclerosis (EAVA) took the responsibility of providing an expert consensus on the future and potential use of these medications in Egyptian patients with dyslipidemia.

## Main text

### The role of low-density lipoprotein cholesterol lowering in atherosclerotic cardiovascular disease prevention

Among all lipid profile parameters, low-density lipoprotein cholesterol (LDL-C) remains the primary analyte and treatment target according to recent guidelines [1]. The causal role of LDL-C in the induction and progression of the atherosclerotic process is unquestionable [2]. Since the introduction of the 3-hydroxymethyl glutaryl co-enzyme A (HMG-co-A) inhibitors [statins] into clinical practice, there has been a robust accumulating evidence that LDL-C lowering results in a reduction of incident ASCVD events (for every 1 mmol/l [39 mg/dl] lowering, there is a corresponding 22% relative risk reduction (RRR) of incident ASCD events) [3]. Moreover, the current emphasis is on the concept that “even the lower LDL-C, even the better”; this is because new evidence coming from the trials of non-statin lipid-lowering therapy points to an additional benefit with further LDL-C lowering beyond target levels previously considered adequate. Ezetimibe (a cholesterol absorption inhibitor) added to simvastatin resulted in a significant 6.4% RRR in the outcome in the IMPROVE-IT trial [4]. Of note, the mean LDL-C achieved in the arm of ezetimibe-simvastatin was 54 mg/dl [4]. The addition of ezetimibe to statins has another potential to reduce the incidence of statin intolerance such as the occurrence of myalgias [5]. Then, the trials of proprotein convertase subtilisin/kexin type 9 (PCSK-9) inhibitors came, which corroborated the notion that lower is better. Evolocumab in the FOURIER trial [6] added to maximally tolerated statin therapy in patients with stable established ASCVD (achieving a mean LDL-C level of merely 30 mg/dl) safely resulted in a significant 15% RRR in a composite ASCVD events endpoint. Alirocumab resulted in a similar magnitude of benefit (with no safety issues) in the ODYSSEY-OUTCOMES trial [7] which examined relatively more unstable patients (those who sustained an acute coronary syndrome (ACS) in the past 12 months). Of note, the ODYSSEY results (in subgroups’ analyses) were consistent among patients with DM and ACS [8] in addition to patients with stroke [9]. Taken together, these data have prompted the 2019 ESC dyslipidemia guideline committee to lower the LDL-C treatment goals across all risk categories, with even a class IIb consideration given to a level of 40 mg/dl in very high-risk patients who are still experiencing ASCVD events [1].

### Reaching the LDL-C goals is difficult

According to the EUROASPIRE-V program [10], which included 8261 patients surveyed after hospitalization for ACS or coronary revascularization in 131 centers covering 27 European countries, about 60% were on high-intensity statins. Only 36% of them achieved an LDL-C level of < 70 mg/dl. The Egyptian data showed that the situation might be worse; among very high-risk patients receiving maximally tolerated statin therapy, LDL-C treatment goals are reached in 22.3% of them [11]. An explanation for these poor figures remains elusive; however, it seems that statins’ effect on LDL-C lowering has a “ceiling effect,” meaning that the highest dose of any statin cannot attain more than 55–60% reduction of baseline LDL-C levels [12]. The problem becomes more complex if we consider that LDL-C treatment goals are becoming furtherly lower in the newer guidelines [1]. This clearly sets the stage for non-statin lipid-lowering therapies to be key players in this field.

### Premature coronary artery disease and familial hypercholesterolemia: current status in Egypt

In recent years, there has been increasing recognition of the problem of familial hypercholesterolemia (FH). The European Atherosclerosis Society (EAS) in collaboration with the Egyptian Association of Vascular Biology and Atherosclerosis (EAVA) and other national societies published an international report about the current status of FH in over 60 countries [13]. Data from the Egyptian CardioRisk project [14] point to a 51% estimated prevalence of premature coronary artery disease (CAD) (defined as ACS before age 55 years in males and 65 years in females) due to noticeably larger burden of traditional risk factors especially smoking and possibly familial hypercholesterolemia. The Egyptian familial hypercholesterolemia research forum is a national initiative powered by EAVA in collaboration with EAS. Its preliminary results [15] showed that among cases with suspected FH (31%, 12%, and 57% in the definite, probable, and possible categories respectively according to the Dutch Lipid Clinic Network criteria), mean total cholesterol was 339 ± 100 mg/dl and mean LDL-C was 249 ± 98 mg/dl. It is imperative to note that these levels are on the background of conventional lipid-lowering therapy (41% statin monotherapy and 59% combination with ezetimibe). Unfortunately, only one patient received lipoprotein apheresis and 6 patients fulfilled for a PCSK-9 inhibitor. The above figures reflect the significant unmet need for a better and a more effective strategy to treat these high-risk patients (including the use of PCSK-9 inhibitors which, after a major price cut [16], are thought now to meet the accepted cost-effectiveness international thresholds [17]).

### Proprotein convertase subtilisin/kexin type 9 becomes recognized

Many reports have revealed that PCSK-9 has a critical role in the life cycle of the LDL receptors [18]. LDL receptors are clustered in clathrin-coated pits on the surface of hepatocytes. After binding to LDL, an endocytic vesicle is produced. In the endosome, the LDL receptor can undergo a conformational change with release of the LDL particle; the LDL receptor is then recycled back to the surface of the hepatocyte to extract more LDL from the circulation. Of note, LDL receptors can be re-circulated approximately 100 times. On the other hand, the LDL particle is transferred to the lysosome where it is degraded. PCSK9 is produced by hepatocytes and attaches to the epidermal growth factor-like repeat A (EGF-A) domain of the LDL receptor. When the PCSK9–LDL receptor complex is internalized, the presence of PCSK9 prevents the conformational change in the LDL receptor, and the LDL receptor then passes together with LDL to the lysosome, where it is destroyed [18].

### PCSK9 monoclonal inhibitors

The advent of monoclonal antibodies against PCSK9 leads the way to a novel mechanism of management of hypercholesterolemia [19]. Evolocumab can be prescribed at a dose of 140 mg every 2 weeks [20]. In phase III clinical trials, evolocumab decreased plasma LDL-C levels by approximately 60% whether given as a monotherapy, added to statin therapy, given to patients who were statin-intolerant, or given to patients with heterozygous FH [21–25]. Overall, evolocumab reduced LDL-C levels regardless of baseline plasma PCSK9 levels [26]. In patients with homozygous FH, the picture was variable. Thus, additive management plans are needed for patients with homozygous FH, especially those with *LDLR*-negative alleles.

Alirocumab has been given with different regimens in phase III clinical trials. One regimen was an escalating dose that was based on attained reductions in plasma LDL-C levels: patients would start at 75 mg of alirocumab every 2 weeks and then the dose would be escalated to 150 mg every 2 weeks if the LDL-C level was ≥ 70 mg/dl. In general, this approach resulted in LDL-C level reductions of 45–50% whether given as monotherapy, added to statin therapy, or given to patients who were statin-intolerant [27–30]. The maximum dose of alirocumab in these trials, 150 mg every 2 weeks, decreased plasma LDL-C levels by about 60% [31], which is like evolocumab. Data concluded that in patients with heterozygous FH, treatment with alirocumab decreased LDL-C levels by about 40–60% [32, 33]. In patients with homozygous FH, the changes in LDL-C levels demonstrated with alirocumab therapy ranged from a 7% rise to a 64% decline according to the genotype of the patient [34].

### Trials that changed the guidelines

The earliest study to report cardiovascular outcomes with the use of PCSK-9 inhibitors was the FOURIER trial [35]. More than 27,000 patients with established atherosclerotic cardiovascular disease (ASCVD) were enrolled in the trial. Patients were eligible for inclusion if they had an LDL-C level ≥ 70 mg/dl or a non-HDL-cholesterol level ≥ 100 mg/dl and taking optimal lipid-lowering therapy, specifically high-intensity statins, with or without ezetimibe. Patients were allocated to receive evolocumab (either 140 mg every 2 weeks or 420 mg every month, according to the patient’s preference) or matching placebo injections. Evolocumab decreased LDL-C levels by 59% relative to placebo, with a mean absolute decrease of 56 mg/dl to a median level of 30 mg/dl, and this reduction was stable over time. Significant reductions (21–27%) were reported in the risks of fatal or nonfatal MI, fatal or nonfatal stroke, and coronary revascularization versus placebo [6]. No impact was observed on cardiovascular death or hospital admissions for unstable angina [6]. As seen in various clinical trials of statins, the translation of LDL-C lowering into clinical benefit is a matter of time. Specifically, the clinical risk reduction per millimoles per liter of LDL-C lowering is much less in the first year of statin therapy compared with the following years [36]. Adverse events tended to be similar between evolocumab and placebo. No higher risk of diabetes was observed with evolocumab relative to placebo even when patients were prediabetics at baseline, which is the subgroup of patients in whom the heightened risk of diabetes with statin therapy seems to be largely confined [37, 38].

When exploring neurocognitive events, the EBBINGHAUS trial was a specific, neurocognitive sub-study conducted on 1974 subjects from the FOURIER trial [39]. No statistically significant differences in cognitive function were noted. Moreover, an exploratory analysis found no consistent correlation between LDL-C levels and cognitive changes [40]. Also, thanks to the fact that evolocumab is a fully human antibody, no patients developed neutralizing antibodies [6].

The ODYSSEY Outcomes trial enrolled a total of 18,924 patients who were recruited 1 to 12 months after hospitalization for MI or unstable angina. Inclusion criteria were LDL-C level ≥ 70 mg/dl, non-HDL-C level ≥ 100 mg/dl, or APOB level ≥ 80 mg/dl. Patients were required to be on high-intensity statin therapy (namely, atorvastatin ≥ 40 mg or rosuvastatin ≥ 20 mg daily) or receiving the maximum tolerable dose of one of those statins. Patients were randomly allocated to receive alirocumab (75 mg or 150 mg every 2 weeks) or matching subcutaneous placebo injections. The dose of alirocumab was escalated to achieve an LDL-C level of 25–50 mg/dl, and alirocumab was halted when the LDL-C level was persistently < 15 mg/dl. The primary efficacy outcome was the composite of CHD death, MI, ischemic stroke, or hospitalization for unstable angina (requiring ECG changes and angiographic evidence of clinically significant disease) [7]. Alirocumab decreased LDL-C levels by 57% at 4 weeks relative to placebo; however, this effect was attenuated with time, with reductions of 50% at 1 year and 36% at the end of the study, due to the down-titration algorithm [7]. The median follow-up was 2.8 years. Over the course of the study, alirocumab showed a significant reduction in the risk of the primary outcome by 15% compared to placebo (HR 0.85, 95% CI 0.78–0.93, *P* = 0.003). The impact of alirocumab on the individual components of the composite endpoint, including coronary heart death, was directionally homogenous, i.e., there was no heterogenous treatment effect. A “nominal” 15% reduction (HR 0.85, 95% CI 0.73–0.98) in all-cause mortality with alirocumab treatment compared with placebo was also noticed. A further analysis according to baseline LDL-C level showed a non-monotonic pattern of risk reduction of the primary efficacy endpoint with alirocumab, with a HR 0.86 (95% CI 0.74–1.01) in the groups of patients with a baseline LDL-C level < 80 mg/dl, HR 0.96 (95% CI 0.82–1.14) in patients with a baseline LDL-C level 80–100 mg/dl, and HR 0.76 (95% CI 0.65–0.87) in patients with a baseline LDL-C level ≥ 100 mg/dl [7]. However, given the idea that the protocol-mandated reduction of therapy on the basis of achieved LDL-C levels, patients assigned to alirocumab treatment who had a lower LDL-C level at baseline were more likely to have their alirocumab dose reduced or stopped, thereby offsetting the useful impact of alirocumab in these patients. The safety data seemed equally reassuring as that seen in the FOURIER trial, with no significant differences in elevations of creatine kinase or aminotransferase levels in the plasma and no excess in the occurrence of new-onset diabetes, cataracts, or neurocognitive declines [7].

## Conclusions

**Fig. 1:**
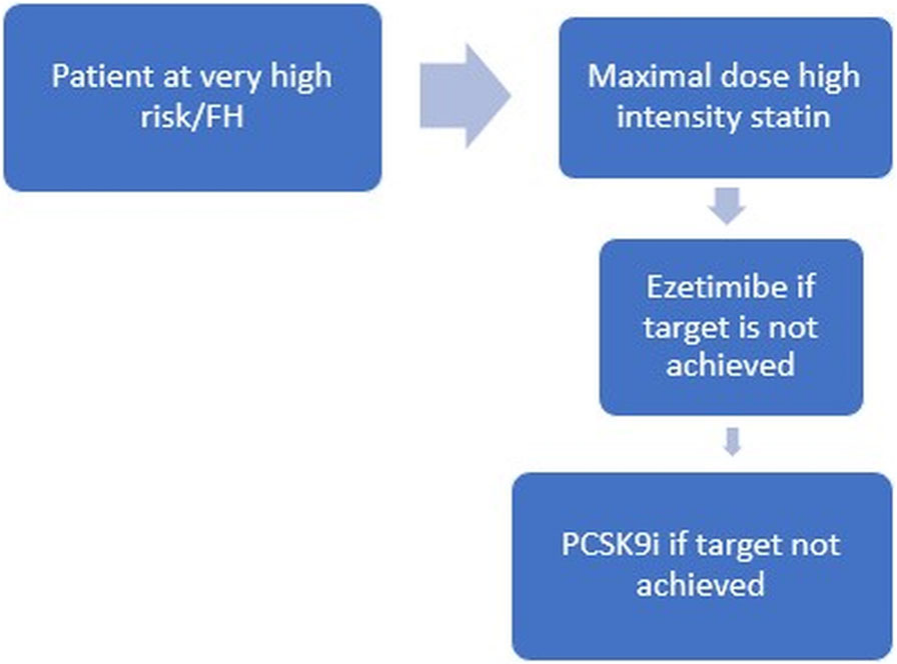
Proposed algorithm for the starting of PCSK9 inhibitors in very high-risk patients and in familial hypercholesterolemia

**Fig. 2:**
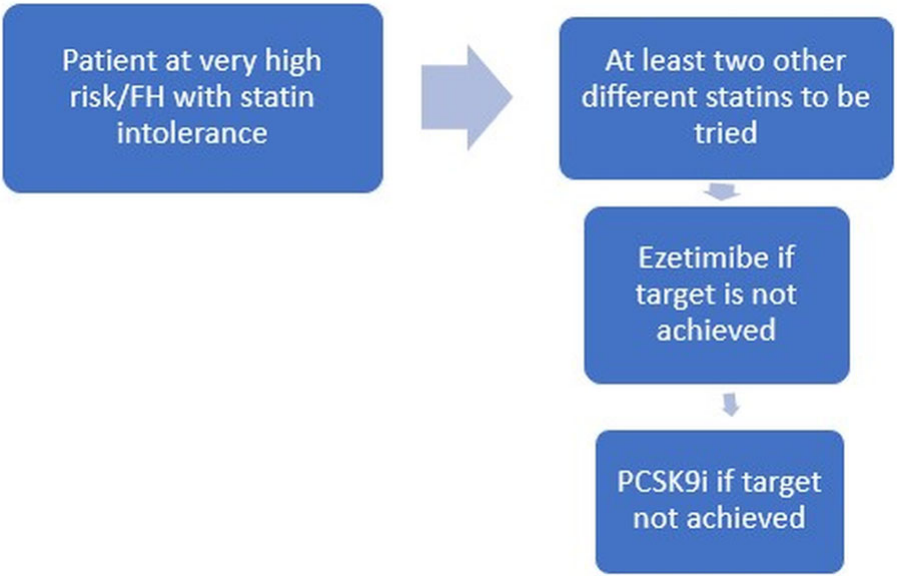
Proposed algorithm for the starting of PCSK9 inhibitors in statin-intolerant patients

Having revised the evidence from the cardiovascular outcome studies with PCSK9 inhibitors as well as from the available Egyptian data as we showed, we can conclude that addition of a PCSK9 inhibitor should be considered in (1) patients with ASCVD, by definition at very high risk (Fig. 1); (2) patients with ASCVD and at very high risk who do not tolerate appropriate doses of at least three statins (Fig. 2); and (3) familial hypercholesterolemia patients with clinically diagnosed ASCVD at high cardiovascular risk or for primary prevention if with very high cardiovascular risk (provided that the patient is on the maximally tolerated statin dose in addition to combination with Ezetimibe, adopting the same targets as in the 2019 ESC guidelines).

## Data Availability

All data are available upon request from the corresponding author(s).

## Abbreviations

ACS: Acute coronary syndrome
ASCVD: Atherosclerotic cardiovascular disease
CAD: Coronary artery disease
EAS: European Atherosclerosis Society
EAVA: Egyptian Association for Vascular Biology and Atherosclerosis
FH: Familial hypercholesterolemia
LDL-C: Low-density lipoprotein cholesterol
PCSK-9: Proprotein convertase subtilisin/kexin type 9
